# Metabolite Profiling, Pharmacokinetics, and In Vitro Glucuronidation of Icaritin in Rats by Ultra-Performance Liquid Chromatography Coupled with Mass Spectrometry

**DOI:** 10.1155/2017/1073607

**Published:** 2017-07-10

**Authors:** Beibei Zhang, Xiaoli Chen, Rui Zhang, Fangfang Zheng, Shuzhang Du, Xiaojian Zhang

**Affiliations:** Department of Pharmacy, The First Affiliated Hospital of Zhengzhou University, Zhengzhou, Henan 450052, China

## Abstract

Icaritin is a naturally bioactive flavonoid with several significant effects. This study aimed to clarify the metabolite profiling, pharmacokinetics, and glucuronidation of icaritin in rats. An ultra-performance liquid chromatography coupled with mass spectrometry (UPLC-MS) assay was developed and validated for qualitative and quantitative analysis of icaritin. Glucuronidation rates were determined by incubating icaritin with uridine diphosphate glucuronic acid- (UDPGA-) supplemented microsomes. Kinetic parameters were derived by appropriate model fitting. A total of 30 metabolites were identified or tentatively characterized in rat biosamples based on retention times and characteristic fragmentations, following proposed metabolic pathway which was summarized. Additionally, the pharmacokinetics parameters were investigated after oral administration of icaritin. Moreover, icaritin glucuronidation in rat liver microsomes was efficient with CL_int_ (the intrinsic clearance) values of 1.12 and 1.56 mL/min/mg for icaritin-3-*O*-glucuronide and icaritin-7-*O*-glucuronide, respectively. Similarly, the CL_int_ values of icaritin-3-*O*-glucuronide and icaritin-7-*O*-glucuronide in rat intestine microsomes (RIM) were 1.45 and 0.86 mL/min/mg, respectively. Taken altogether, dehydrogenation at isopentenyl group and glycosylation and glucuronidation at the aglycone were main biotransformation process in vivo. The general tendency was that icaritin was transformed to glucuronide conjugates to be excreted from rat organism. In conclusion, these results would improve our understanding of metabolic fate of icaritin in vivo.

## 1. Introduction

Herba Epimedii, the dried aerial parts of* Epimedium* L. (Berberidaceae), are a widely used Chinese medicine for impotence, bone loss, and cardiovascular diseases [1–3]. Prenylflavonoids are reported to be a group of major active constituents present in* Epimedium* for the antioxidative stress, anti-inflammatory, antitumor, and antiosteoporosis activities [4–8]. Icaritin is the common aglycone with many biological effects, especially antiosteoporosis activities [5, 7]. Besides, icaritin could induce cell death in activated hepatic stellate cells through mitochondrial activated apoptosis and ameliorate the development of liver fibrosis in rats [9]. Meanwhile, icaritin is able to target androgen receptor and androgen receptor COOH-terminal truncated splice variants, to inhibit androgen receptor signaling and tumor growth with no apparent toxicity [10]. Additionally, icaritin has neuroprotective effects against MPP^+^-induced toxicity in MES23.5 cells. IGF-I receptor mediated activation of PI3K/Akt and MEK/ERK1/2 pathways are involved in the neuroprotective effects of icaritin against MPP^+^-induced neuronal damage [11]. Recently, icaritin had been shown as a potential agent for the treatment of systemic lupus erythematosus [12].

These biological activities above had stimulated increasing interests in the in vivo metabolism of icaritin or its related prenylflavonoids. Poor bioavailability of prenylated flavonoids results from their poor intrinsic permeation and transporter-mediated efflux by the human intestinal Caco-2 model and the perfused rat intestinal model [13]. Meanwhile, it is shown that Epimedium flavonoids could be hydrolyzed into secondary glycosides or aglycone by intestinal flora or enzymes, thereby enhancing their absorption and antiosteoporosis activity [14]. So far, numerous researches of total prenylflavonoids or individual flavonoid had been conducted in the fields of in vivo metabolites profiling, biliary excretion, and pharmacokinetics [15–19]. Generally, the in vivo metabolism of* Herba Epimedii *extracts or its prenylflavonoids could easily be metabolized in gastrointestinal tract following deglycosylation reaction. Additionally, icaritin was easily metabolized into glucuronidation conjugates to be preferentially eliminated and excreted from rat organism [16, 18, 20]. Though the data on metabolic researches of icaritin abounds, its metabolic profile is not so clear. It is essential to systematically characterize the in vivo metabolites in order to better understand its mechanism of action. Hence, the present study aimed to conduct the metabolites screening, quantitative determination, and in vitro glucuronidation of icaritin.

Recently, liquid chromatography coupled with mass spectrometry (LC-MS) had been widely introduced to rapidly screen trace components in biological samples [21, 22]. In this study, icaritin-related metabolites were analyzed based on characteristic fragmentation by UPLC-MS after oral administration. Meanwhile, possible disposing pathway of icaritin was proposed. Furthermore, a UPLC-MS method was developed and applied to perform the pharmacokinetics of icaritin. Moreover, glucuronidation rates were determined by incubating icaritin with uridine diphosphate glucuronic acid- (UDPGA-) supplemented rat liver microsomes (RLM) and rat intestine microsomes (RIM). Kinetic parameters were derived by appropriate model fitting. Icaritin was subjected to significant hepatic and gastrointestinal glucuronidation.

## 2. Materials and Methods

### 2.1. Materials

Icaritin, epimedin C, icariside I, icariside II, and desmethylicaritin (purity > 98%) were purchased from Nanjing Jingzhu Medical Technology Co., Ltd. Uridine diphosphate glucuronic acid (UDPGA), magnesium chloride (MgCl_2_), alamethicin, D-saccharic-1, and 4-lactone were provided from Sigma-Aldrich (St. Louis, MO). Rat liver microsomes (RLM) and rat intestine microsomes (RIM) were prepared in our laboratory based on the protocol [21]. HPLC grade methanol and acetonitrile were purchased from Dikma Scientific and Technology Co., Ltd. All other chemicals were of analytical grade.

### 2.2. Animals

Male Sprague-Dawley rats (180~220) g were provided by Guangdong Medical Laboratory Animal Center. The rats were kept in an animal room at constant temperature (24 ± 2)°C and humidity (60 ± 5)% with 12 h of light/dark per day and free access to water and food. The animal protocols were approved and conducted in accordance with the guidelines of Laboratory Animal Ethics Committee of Zhengzhou University.

### 2.3. Samples Collection and Preparation for Qualitative Analysis

After the rats were fasted for 12 h with free access to water before experiments, icaritin dissolved in 0.3% sodium carboxymethyl cellulose solution was orally administrated to rats at a dose of 100 mg/kg. Blood samples were collected from external jugular vein into heparinized tubes and were separated by centrifuging at 13800*g* for 10 min at 4°C, respectively. Bile samples were collected and recorded during 0–24 h period after an abdominal incision anesthetized with 10% aqueous chloral hydrate. The urine and feces samples were collected separately during 0–24 h period after oral administration. Small intestinal samples were obtained after oral administration for 24 h. All blank samples were obtained in the same way.

Before experiments, all biosamples were stored at −20°C. In this work, solid phase extraction method was applied to pretreat all samples. Before use, C18 columns (3 cm^3^, 60 mg) were first preconditioned and equilibrated with 3 mL of methanol and 3 mL of water, respectively. Urine samples were evaporated and concentrated at 40°C under reduced pressure. Feces samples and small intestinal samples were dried in air and stirred into powder. And then they were treated with an ultrasonic bath for 30 min. The filtrate was combined and evaporated to dryness at 40°C in vacuum. The residue was reconstituted with water. Plasma, urine, bile, feces, and small intestinal samples were loaded on pretreated columns. The residue was reconstituted in 200 *μ*L of 60% methanol and filtered through a 0.22 *μ*m membrane until injection.

### 2.4. Samples Preparation for Quantitative Analysis

Plasma sample (200 *μ*L) was treated with methanol (1.2 mL), after which the mixture was vortex-mixed for 30 s and centrifuged at 13800*g* for 10 min at 4°C. The supernatant was then transferred and evaporated to dryness using N_2_ at room temperature. The residue was dissolved in 200 *μ*L of 60% methanol and was then injected into the UPLC-MS system.

### 2.5. Preparation of Standard Solutions

Blank rat plasma was spiked with standard working solutions to achieve final concentration of icaritin of 2.0, 4.0, 16.0, 64.0, 128.0, 256.0, and 512.0 ng/mL. All reference standard solutions were stored at 4°C until use.

### 2.6. Glucuronidation Assay

Icaritin was incubated with RLM and RIM to determine the rates of glucuronidation as published references previously [23]. Briefly, the incubation mixture mainly contained 50 mM Tris-hydrochloric acid buffer (pH = 7.4), 0.88 mM MgCl_2_, 22 *μ*g/mL alamethicin, 4.4 mM saccharolactone, and 3.5 mM UDPGA. The reaction was terminated by adding ice-cold acetonitrile. The samples were vortexed and centrifuged at 13800*g* for 10 min. The supernatant was subjected to UPLC-MS analysis. All experiments were performed in triplicate.

### 2.7. UPLC-MS Conditions

UPLC was performed using an ACQUITY™ UPLC system (Waters, Milford, MA, USA). Separation was achieved on a Waters BEH C18 column (1.7 *μ*m, 2.1 × 50 mm) maintained at 35°C. The mobile phase consisted of water (A) and acetonitrile (B) (both containing 0.1% formic acid), and the flow rate was 0.5 mL/min. The gradient elution program was as follows: 0 min, 15% B; 3 min 35% B; 7 min 60% B; 8 min 100% B. An aliquot of 4 *μ*L sample was then injected into the UPLC-MS system.

The UPLC system was coupled to a Waters Xevo TQD (Waters, Milford, MA, USA) with electrospray ionization. The operating parameters were as follows: capillary voltage, 2.5 kV (ESI+); sample cone voltage, 30.0 V; extraction cone voltage, 4.0 V; source temperature, 100°C; desolvation temperature, 300°C; and desolvation gas flow, 800 L/h. The method employed lock spray with leucine enkephalin (*m/z* 556.2771 in positive ion mode and* m/z* 554.2615 in negative ion mode) to ensure mass accuracy.

### 2.8. Pharmacokinetic Application

After fasting with free access to water for 12 h, icaritin was given to rats as a dosage of 100 mg/kg. Plasma samples were then obtained at 0.083, 0.25, 0.5, 1, 2, 3, 4, 6, 8, 12, 24, 36, and 48 h after administration. For pharmacokinetic application, DAS 2.0 was used to calculate the pivotal pharmacokinetic parameters.

### 2.9. Enzymes Kinetic Evaluation

Serial concentrations of icaritin (0.4~20 *μ*M) were incubated with RLM and RIM to determine icaritin glucuronidation rates. The kinetic models Michaelis-Menten equation and substrate inhibition equation were fitted to the data of metabolic rates versus substrate concentrations and displayed in (1) and (2), respectively. Appropriate models were selected by visual inspection of the Eadie-Hofstee plot [24]. Model fitting and parameter estimation were performed by Graphpad Prism V5 software (San Diego, CA).

The parameters were as follows. *V* is the formation rate of product. *V*_max_ is the maximal velocity. *K*_m_ is the Michaelis constant and [*S*] is the substrate concentration. *K*_si_ is the substrate inhibition constant. The intrinsic clearance (CL_int_) was derived by *V*_max_/*K*_m_ for Michaelis-Menten and substrate inhibition models.

(1) V=Vmax×SKm+S,

(2) V=Vmax×SKm+S1+S/Ksi.

## 3. Results

### 3.1. Fragment Pattern of Icaritin

As had already been reported in the previous study [16], besides the typical adduct ion [M+Na]^+^ at* m/z *391.1151 (C_21_H_20_O_6_Na) and [M+H]^+^ at* m/z* 369.1336 (C_21_H_21_O_6_, −0.5 ppm), the ion at* m/z *313.0714 (C_17_H_13_O_6_) in positive ion mode was considered as the characteristic fragment ion (see Figure S1a in the Supplementary Material available online at https://doi.org/10.1155/2017/1073607).

### 3.2. Screening of Metabolites

**Figure 1:**
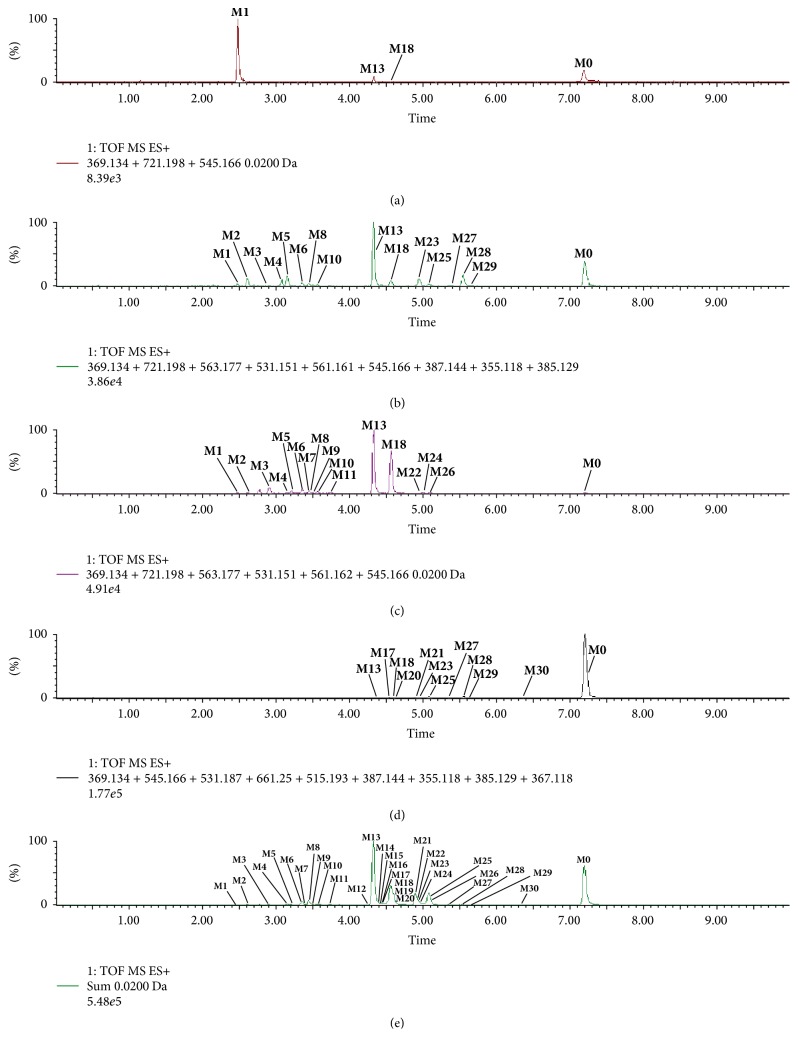
EICs of all metabolites in rat biosamples after oral administration of icaritin. (a) Plasma; (b) urine; (c) bile; (d) feces; (e) intestine.

On the basis of MS/MS fragmentation pattern, the metabolites were deduced, clarifying the general metabolism in vivo. The extracted ion chromatograms (EICs) of prototype (M0) and metabolites (M1~M30) were shown in Figure 1, while the individual EICs of M1~M30 were exhibited in Figure S2. The UV, MS, and MS/MS data of M0~M30 were all exhibited in Table 1.

### 3.3. Structure Elucidation of Metabolites

*M0 (Parent Drug).* M0 (7.20 min, C_21_H_20_O_6_, −0.5 ppm) in biological samples was unambiguously identified by comparing with references. 

*M15 and M23 (Hydration of Isopentene Group).* Based on the [M+H]^+^ ion at* m/z* 387.1445 (C_21_H_23_O_7_, 0.3 ppm) and [M−H]^−^ ion at* m/z* 385.1286 (C_21_H_21_O_7_), the molecular formula of M15 (4.34 min) and M23 (4.97 min) was determined as C_21_H_22_O_7_, with one H_2_O more than M0. The MS/MS spectrum of [M+H]^+^ ion (C_21_H_23_O_7_) showed predominant [M+H−H_2_O]^+^ ion at* m/z* 369.1343 and 313.0715 (Figure S1b), which indicated that M15 and M23 were the hydration products at isopentene group of icaritin and agreed with previous study of icariin [18]. 

*M25 (Demethylation of Flavonoid Aglycone)*. According to the [M+H]^+^ ion at* m/z* 355.1180 (C_20_H_19_O_6_, −0.6 ppm) and [M−H]^−^ ion at* m/z *353.1036 (C_20_H_17_O_6_), the formula of M25 (5.08 min) was supposed as C_20_H_18_O_6_, with a methyl group less than M0. The MS/MS experiments (Figure S1c) showed a significant loss of neutral loss of C_4_H_8_ (56.0626 Da) from the ion at* m/z* 355.1180 to 299.0582 in positive ion mode or from the ion at* m/z* 353.1036 to 297.0373 in negative ion mode. Meanwhile, the demethylation position was purposed at 4′ position of B ring of flavonoid aglycone. Moreover, M25 was identified as desmethylicaritin by comparison of reference standard. 

*M30 (Dehydrogenation of Isopentene Group)*. The formula of M30 (6.36 min) was C_21_H_18_O_6_, with two hydrogens fewer than M0, based on the [M+H]^+^ ion at* m/z* 367.1180 (C_21_H_19_O_6_, −0.5 ppm) and [M−H]^−^ ion at* m/z *365.1047 (C_21_H_17_O_6_). In MS/MS spectrum (Figure S1d), the ions at* m/z* 352.0948 and 313.0721 were attributed to obvious loss of CH_3_ (15.0235 Da) and C_4_H_6_ (54.0470 Da) group, respectively, which indicated that the dehydrogenation position was at isopentene group [18].

*M27~M29 (Hydroxylation of Isopentene Group)*. From the [M+H]^+^ ion at* m/z* 385.1288 (C_21_H_21_O_7_, 0.3 ppm) and [M−H]^−^ ion at* m/z *383.1175 (C_21_H_19_O_7_), the formulae of M27 (5.37 min, *λ*_max_ 268 nm), M28 (5.54 min), and M29 (5.66 min) were speculated as C_21_H_20_O_7_, which was one oxygen more than M0. In (+) ESI-MS/MS spectrum (Figure S1e), the ion at* m/z* 385.1288 could lose a H_2_O and C_4_H_6_ group to produce the daughter ions at 367.1186 ([M+H−H_2_O]^+^) and 313.0715 ([M+H−C_4_H_6_]^+^), respectively. This illustrated that M27~M29 were tentatively characterized as the hydroxylated products of M0 at the isopentene group. 

*M14, M16, M17, M19, M20, and M21 (Glycosylation of Flavonoid Aglycone)*. M21 (4.88 min) was given a [M+H]^+^ ion at* m/z* 515.1927 (C_27_H_31_O_10_, 1.9 ppm) and [M−H]^−^ ion at* m/z *513.1766 (C_27_H_29_O_10_) in full scan mass spectrum. The ion at* m/z* 515.1927 could easily yield the characteristic fragment ions at* m/z* 369.1340 and 313.0726 by subsequent loss of C_6_H_10_O_4_ and C_4_H_8_ (Figure S1f). So M21 could be the glycosylation product of M0 by adduct of rhamnose (C_6_H_10_O_4_, 146.0579 Da). Similarly, M17 (4.53 min, C_27_H_30_O_11_, 1.5 ppm) was the glucose conjugate of M0, while M19 (4.59 min, C_32_H_39_O_14_, 1.4 ppm) and M20 (4.61 min, C_33_H_41_O_14_, 1.1 ppm) were the xylose and rhamnose glycosylation derivates of M21, respectively. M14, M17, and M21 were identified as epimedin C, icariside I, and icariside II, respectively.

M14 (4.40 min, *λ*_max_ 270 nm, C_39_H_51_O_19_, −0.1 ppm) and M16 (4.49 min, *λ*_max_ 270 nm, C_39_H_51_O_19_, −0.5 ppm) both with the formula of C_39_H_50_O_19_ (Figure S1g) were tentatively characterized as the glucose glycosylation conjugate of M20. These glycosylation reactions were the same as the metabolism of epimedin C in rats reported in reference (Liu et al., 2011). By comparing with references, M14, M17, and M21 were identified as epimedin C, icariside I, and icariside II, respectively. And the MS/MS spectra of M17, M19, and M20 were shown in Figures S1h–S1j, respectively.

*M1, M8, M13, M18, and M26 (Glucuronidation of Flavonoid Aglycone)*. In full scan mass spectrum, M13 (4.34 min), M18 (4.57 min), and M26 (5.10 min) all exhibited the [M+H]^+^ ion at* m/z* 545.1663 (C_27_H_29_O_12_, 0.7 ppm) and [M−H]^−^ ion at* m/z *543.1501 (C_27_H_27_O_12_) with a formula of C_27_H_28_O_12_ of 176.0325 Da larger than M0. The MS/MS spectrum (Figure S1k) displayed an obvious loss of C_6_H_8_O_6_ group from parent ion at* m/z* 545.1663 to the daughter ion at* m/z *369.1338, which suggested an existing glucuronic acid of these three metabolites. Just like reported studies [16], monoglucuronide conjugate and diglucuronide conjugate were widely distributed in biological samples after oral administration of Epimedium-related total flavonoids or individual flavonoid. Therefore, M13, M18, and M26 were tentatively identified as monoglucuronidation conjugate of M0, while M1 (2.48 min, C_33_H_36_O_18_, −0.3 ppm) and M8 (3.45 min, C_33_H_36_O_18_, 0.3 ppm) (Figure S1l) were characterized as diglucuronidation derivates based on two molecules of C_6_H_8_O_6_ fragment larger than M0.

Similarly, M2 (2.62 min, *λ*_max_ 269 nm, C_27_H_30_O_13_, 0.5 ppm) and M10 (3.57 min, *λ*_max_ 269 nm, C_27_H_30_O_13_, −0.7 ppm) with the MS/MS spectrum shown in Figure S1 m were tentatively considered as the monoglucuronidation products of M15 and M23. M3 (2.89 min, *λ*_max_ 345 nm, C_26_H_26_O_12_, 1.5 ppm) and M6 (3.36 min, *λ*_max_ 345 nm, C_26_H_26_O_12_, 0 ppm) were characterized as monoglucuronide conjugate of M25. The MS/MS spectrum of M3 and M6 was exhibited in Figure S1n. Meanwhile, M4 (3.16 min, *λ*_max_ 341 nm, C_27_H_28_O_13_, 1.6 ppm), M5 (3.21 min, *λ*_max_ 341 nm, C_27_H_28_O_13_, 0.9 ppm), M7 (3.40 min, *λ*_max_ 341 nm, C_27_H_28_O_13_, −0.5 ppm), M9 (3.50 min, *λ*_max_ 341 nm, C_27_H_28_O_13_, 0 ppm), and M11 (3.74 min, *λ*_max_ 341 nm, C_27_H_28_O_13_, 1.2 ppm) were regarded as monoglucuronidation derivates of M27~M29 and their MS/MS spectrum was displayed in Figure S1o. M12 (4.29 min, not available *λ*_max_, C_27_H_26_O_12_, −0.4 ppm), M22 (4.94 min, *λ*_max_ 300 nm, C_27_H_26_O_12_, −0.6 ppm), and M24 (5.02 min, *λ*_max_ 300 nm, C_27_H_26_O_12_, 0.4 ppm) were tentatively identified as glucuronidation conjugates of M30. And their MS/MS spectrum was shown in Figure S1p.

### 3.4. Method Validation

The method was validated for specificity, linearity, extraction recovery, matrix effects, precision, accuracy, and stability according to the US Food Drug Administration guidelines for bioanalytical method validation [25].

Specificity was determined by comparing the chromatograms obtained for six blank plasma samples, blank plasma samples spiked with standard solutions at LLOQ concentrations, and drug plasma samples obtained 4 h after oral administration. As shown in Figure S3, no interference peaks were detected at the retention times of icaritin.

The LOD and LOQ were calculated as 3-fold and 10-fold of the ratio of signal-to-noise, respectively. The LLOQ was defined as the lowest concentration in the calibration curve with accuracy of 80~120% and precision of 20%. Calibration curves were acquired by plotting peak area (*y*) versus respective plasma concentrations (*x*) using a 1/*x*^2^ weighting factor and linear least-squares regression analysis. A series of standard solutions were used to generate calibration curve. The correlation coefficients (*r*^2^) of calibration curves were greater than 0.9926 within 2.0~512.0 ng/mL and LLOQ was 2.0 ng/mL. The regression equations, correlation coefficients, and LLOQ were shown in Table S1.

The experiments to evaluate matrix effect and recovery were conducted by the protocol [26]. According to the protocol, the peak areas from QC samples at three concentrations were defined as A1; those from extracted control plasma reconstituted with standard solutions at 4.0, 64.0, and 256.0 ng/mL were A2. The responses of icaritin found by direct injection of the corresponding pure reference standards at three QC levels were A3. The matrix effect and recovery were calculated as follows: matrix effect (%) = A2/A3 × 100%. Recovery (%) = A1/A2 × 100%. The results (as shown in Table S2) illustrated that matrix effect was between 89.1% and 113.5%, and the recovery was from 96.3% to 102.7%.

The accuracy and inter/intraday precision of the method were evaluated by determining six replicates of QC samples on three consecutive days. The measured concentrations of QC samples were determined with a calibration curve obtained on the same day. Relative error and relative standard deviation were used to describe accuracy and inter/intraday precision, respectively. They both should not exceed 15%. As exhibited in Table S3, the intraday and interday precision were less than 13.2% and 10.2%, respectively, while the intraday and interday precision of LLOQ were no more than 17.4% and 15.6%, respectively.

Stability of icaritin in rat plasma was assessed under different conditions at three concentration levels, including extracted samples for 12 h at room temperature, kept at −20°C for 60 h, three cycles of freezing at −20°C and thawing at 25°C, and plasma sample at room temperature for 8 h. Each was compared by three QC replicates of the same concentration with a calibration curve in the same day. The RE was within 13.8% and RSD was less than 11.3%. Stability results (Table S4) indicated that icaritin were stable under different storage conditions.

### 3.5. Pharmacokinetics Application

**Figure 2:**
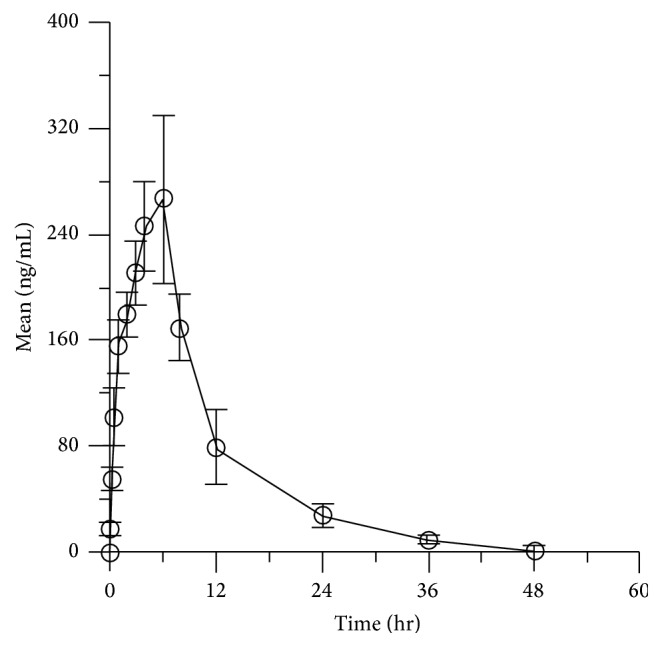
Concentration-time curve of icaritin in rat plasma after oral administration.

The mean concentration-time profiles of these bioactive components were shown in Figure 2. The main pharmacokinetic parameters were illustrated in Table 2. In this study, *C*_max_ was (294.5 ± 22.7) ng/mL when *T*_max_ was (5.3 ± 1.1) h after oral administration. The area under the concentration-time curve (AUC_0−*∞*_) and mean residence time (MRT_0-∞_) were (3145.0 ± 302.3) ng·h/mL and (10.9 ± 1.3) h, respectively. The results illustrated that icaritin had a poor absorption after oral administration. The reason may be that icaritin stepped into small intestine to undergo mass phase I and phase II metabolism by intestinal flora, especially the glycosylation and glucuronidation conjugates.

### 3.6. Glucuronidation of Icaritin in RLM and RIM

Due to lack of reference standard, quantification of icaritin glucuronide was based on the standard curve of the parent compound (icaritin) according to the assumption that parent compound and its glucuronide have closely similar UV absorbance maxima [27–29]. The detection wavelength of icaritin and icaritin glucuronides was 270 nm. The linear range of icaritin was 0.02~20 *μ*M, with LOD (*S*/*N* = 3~5) and LOQ (*S*/*N* = 8~10) of 0.01 and 0.02 *μ*M, respectively. And the acceptable linear correlation (*Y* = 12149*X*) was confirmed by correlation coefficients (*r*^2^) of 0.9994. The accuracy and precision of the intraday and interday error were both less than 3.4%. There were no matrix effects observed and no other sample preparation performed except those mentioned in the manuscript.

**Figure 3:**
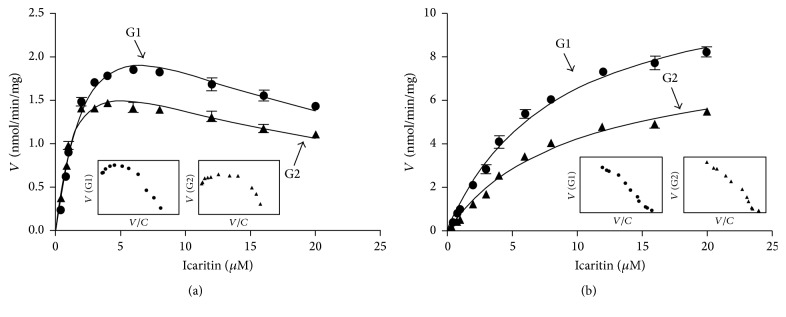
Kinetic profiles for glucuronidation of icaritin by various types of microsomes. (a) Pooled rat liver microsomes (RLM); (b) pooled rat intestine microsomes (RIM). In each panel, the insert figure showed the corresponding Eadie-Hofstee plot.

Kinetic profiling revealed that formation of icaritin-3-*O*-glucuronide (M13) and icaritin-7-*O*-glucuronide (M18) in RLM was well modeled by the substrate inhibition equation (Figure 3(a)), whereas they followed the classical Michaelis-Menten kinetics in RIM (Figure 3(b)). In contrast, the glucuronide formation of M13 (4.06 nmol/min/mg) and M18 (2.39 nmol/min/mg) in RLM was similar as well as M13 (11.88 nmol/min/mg) and M18 (8.23 nmol/min/mg) in RIM. Icaritin glucuronidation in RLM was efficient (CL_int_ = 1.12 and 1.56 mL/min/mg for M13 and M18, resp.), following the substrate inhibition kinetics with *K*_m_ values of 3.62 and 1.53 *μ*M, respectively. Similarly, the CL_int_ values of M13 and M18 in RIM were 1.446 and 0.861 mL/min/mg, respectively, whereas the *K*_m_ values of M13 and M18 in RIM in Michaelis-Menten model were 8.22 and 9.56 *μ*M, respectively. In addition, *K*_i_ values of M13 and M18 in RLM were 11.31 and 17.07 *μ*M, respectively. The detailed parameters of M13 and M18 were listed in Table 3.

## 4. Discussion

Normally, only the prototypes or metabolites in blood with a high enough exposure in target organs for a finite period of time are considered as potential effective components for therapeutic benefits [30]. In this study,** M0**,** M1**, and** M13** were the main xenobiotics in plasma (Figure 1(a)), which may be the potential in vivo effective components directly. After circulation**, M2**,** M5**,** M13**,** M23**, and** M28 **were passed out with the urine (Figure 1(b)).

Due to poor oral bioavailability, several components were limited to be absorbed in blood. But they could influence intestinal dysfunction to exert efficacy by their prototypes, secondary metabolites, or finally the aglycone in intestinal tract [31]. Massive metabolites containing** M6, M8, M13, M17, M25, M28,** and** M30** were detected in rat feces and small intestinal samples (Figure 1(d)). Moreover, icaritin underwent phase II metabolism by main conjugating enzymes including UDP-glucuronosyltransferases (UGTs) to produce extensive mono- or diglucuronic acid conjugates. In rat bile,** M3, M6, M13, M18,** and** M24** mainly were biotransformed in rat liver and excreted into bile (Figure 1(c)).

Characterization of icaritin glucuronidation assumed a great role in the understanding of its pharmacokinetics and bioavailability. Oral bioavailability is a major factor in determining the biological actions of icaritin in vivo following oral administration of the compound [32]. This study suggested that the oral bioavailability of icaritin would be influenced by first-pass glucuronidation in the liver. The glucuronidation activity was obtained by kinetic profiling and modeling. Kinetic profiling required the determination of the rates of icaritin glucuronidation at a series of icaritin concentrations. The relative activities of RLM and RIM toward icaritin glucuronidation were evaluated by the derived CL_int_ values (Table 3). Use of CL_int_ (=*V*_max_/*K*_m_) as an indicator of enzymes activity was advantageous, because (1) CL_int_ represents the catalytic efficiency of the enzyme and is independent of the substrate concentration; (2) compared with other kinetic parameters such as *K*_m_ and *V*_max_, CL_int_ is more relevant in an attempt to predict hepatic clearance in vivo [33]. Therefore, CL_int_ values were used to determine icaritin glucuronidation activity in this study.

**Figure 4:**
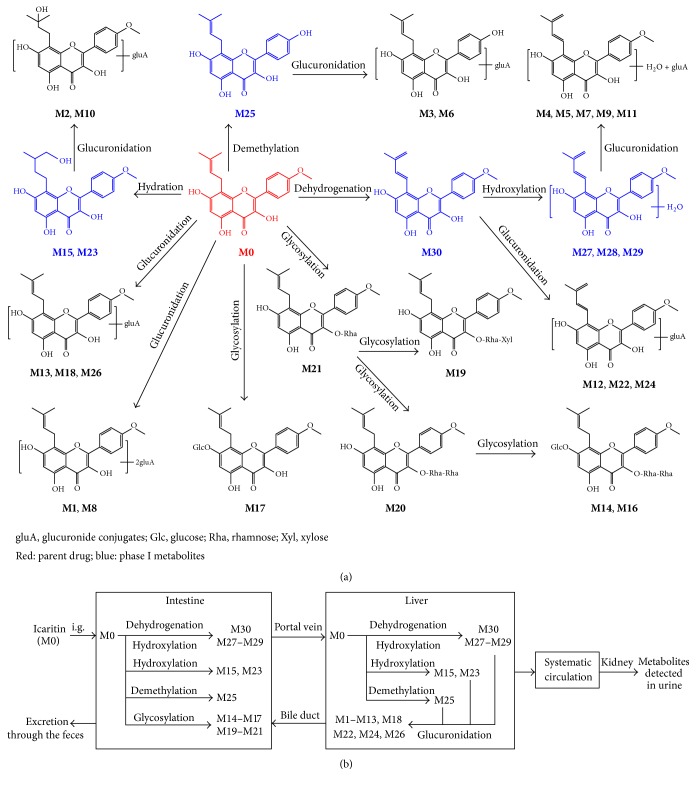
The proposed metabolic pathway (a) and metabolic sites (b) of icaritin in rats.

Based on the metabolite profiles, the metabolic pathways of icaritin were proposed and shown in Figure 4(a), and the metabolic sites were shown in Figure 4(b). In summary, icaritin was hard to be absorbed into the rat blood. In small intestine, icaritin could form flavonoid glycoside by the sequential glycosylation metabolism. Meanwhile, icaritin could easily conjugate with a glucuronic acid to form phase II metabolites in liver, which indicated that the biliary clearance was one of the major routes of excretion. Phase I metabolism of icaritin mainly included demethylation, dehydrogenation, and hydration. The general tendency was that the saponins were metabolized and transformed into the high polar metabolites to be eliminated and excreted from the rat organism.

## 5. Conclusion

As a result, a total of 30 metabolites were identified or tentatively characterized based on the retention time behaviors and fragmentation patterns. Dehydrogenation at isopentenyl group and glycosylation and glucuronidation at the flavonoid aglycone were the main biotransformation process of icaritin in vivo. Meanwhile, a validated method was successfully applied to a pharmacokinetic study. Moreover, icaritin glucuronidation in RLM was efficient with CL_int_ values of 1.12 and 1.56 mL/min/mg for M13 and M18, respectively. Similarly, the CL_int_ values of M13 and M18 in RIM were 1.45 and 0.86 mL/min/mg, respectively. Taken altogether, this study could provide an experimental basis to understand the metabolic fate of icaritin in rat.

## Supplementary Material

Table S1: Leaner range and LLOQ test of icaritin in rat plasma. Table S2: Matrix effect and recovery test of icaritin in rat plasma (*n* = 6). Table S3: Intra- and inter-day precision and accuracy test of icaritin in rat plasma. Table S4: Stability test of icaritin in rat plasma under different condition (*n* = 3). Figure S1: (+) ESI-MS and MS/MS spectra of M0~M30. Figure S2: EICs of M0~M30 in rat intestine samples. Figure S3: Specificity test of icaritin in rat plasma.

## Figures and Tables

**Table 1:** UPLC-MS analysis of icaritin and its observed metabolites in biosamples.

Number	*t*_*R*_	UV	[M + H]^+^	Error	Formula	(+) ESI-MS/MS	(–) ESI-MS/MS	Sources	Characterization
	min	nm		ppm					
M0^#^	7.20	272	369.1336	–0.5	C_21_H_20_O_6_	391.1152 **369.1336 **313.0714 215.0748	**367.1180** 352.0941 309.0417 297.0399 281.0443 253.0485	PUBFI	Icaritin
M1	2.48	270	721.1978	–0.3	C_33_H_36_O_18_	743.1882 721.1978 545.1666 **369.1345** 313.0717	719.1825 543.1521 **367.1194**	PUBI	Icaritin-di-*O*-gluA
M2	2.62	269	563.1768	0.5	C_27_H_30_O_13_	563.1768 **387.1442 **369.1332 313.0716	561.1608 **385.1288**	UBI	Hydrated icaritin-*O*-gluA
M3	2.89	345	531.1511	1.5	C_26_H_26_O_12_	531.1511 **355.1166** 299.0576	529.1348** 353.1027 **119.0504	UBI	Desmethylicaritin-*O*-gluA
M4	3.15	341	561.1617	1.6	C_27_H_28_O_13_	561.1617 **385.1301** 367.1186	559.1456 **383.1175** 367.1135 175.0263	UBI	Hydroxylated icaritin-*O*-gluA
M5	3.22	341	561.1613	0.9	C_27_H_28_O_13_	561.1613 **385.1296**	559.1458 **383.1172** 175.0262	UBI	Hydroxylated icaritin-*O*-gluA
M6	3.36	345	531.1503	0.0	C_26_H_26_O_12_	531.1503 **355.1175 **329.0741 314.0455	529.1345 **353.1025** 119.0506	UBI	Desmethylicaritin-*O*-gluA
M7	3.41	341	561.1605	–0.5	C_27_H_28_O_13_	561.1605 **385.1295 **313.0715	559.1460 **383.1171**	BI	Hydroxylated icaritin-*O*-gluA
M8	3.46	270	721.1982	0.3	C_33_H_36_O_18_	721.1982 545.1662 **369.1340** 313.0717	719.1823 543.1519 **367.1196**	UBI	Icaritin-di-*O*-gluA
M9	3.52	341	561.1608	0	C_27_H_28_O_13_	561.1608** 385.1283 **	559.1454 **383.1170** 175.0260	BI	Hydroxylated icaritin-*O*-gluA
M10	3.58	269	563.1761	–0.7	C_27_H_30_O_13_	563.1761 **387.1452 **369.1350 313.0735	561.1605 **385.1289**	UBI	Hydrated icaritin-*O*-gluA
M11	3.74	341	561.1615	1.2	C_27_H_28_O_13_	561.1615 **385.1292** 367.1186	559.1462 **383.1175**	BI	Hydroxylated icaritin-*O*-gluA
M12	4.29	n.a.	543.1501	–0.4	C_27_H_26_O_12_	543.1501 **367.1189 **	541.1343 **365.1034 **351.0765 175.0221 113.0253	I	Dehydrogenated icaritin-*O*-gluA
M13	4.34	271	545.1663	0.7	C_27_H_28_O_12_	567.1486 545.1663 **369.1335 **313.0716	543.1501** 367.1188** 352.0948 309.0408 297.0398 281.0498	PUBFI	Icaritin-*O*-gluA
M14^#^	4.40	270	823.3024	–0.1	C_39_H_50_O_19_	823.3024** 369.1324** 313.0721	821.2863 659.2331 **367.1168 **351.0867	I	Epimedin C
M15	4.45	273	387.1440	–1.0	C_21_H_22_O_7_	**387.1440 **369.1338 313.0722	**385.1288 **	I	Hydrated icaritin
M16	4.49	270	823.3021	–0.5	C_39_H_50_O_19_	823.3021 **369.1336 **313.0725	821.2860 659.2333 **367.1165 **351.0869	I	Epimedin C isomer
M17^#^	4.53	270	531.1866	1.5	C_27_H_30_O_11_	531.1866 **369.1350** 313.0718	529.1705 **367.1182** 352.0948 297.0395 253.0496	FI	Icariside I
M18	4.57	271	545.1661	0.4	C_27_H_28_O_12_	545.1661 **369.1346 **313.0723	543.1505** 367.1183** 352.0945 297.0394	PUBFI	Icaritin-*O*-gluA
M19	4.59	271	647.2349	1.4	C_32_H_38_O_14_	669.2215 647.2349 515.1930 **369.1346** 313.0724	645.2168** 367.1162 **351.0863 323.0945 295.0595	I	Icaritin-rha-xyl
M20	4.61	270	661.2503	1.1	C_33_H_40_O_14_	683.2326 661.2503 **369.1339** 313.0719	659.2343 **367.1165** 351.0865 295.0602 217.0499	FI	Icaritin-rha-rha
M21^#^	4.88	269	515.1927	1.9	C_27_H_30_O_10_	537.1757 515.1927** 369.1340** 313.0726	513.1766 **367.1157** 352.0900 323.0910 295.0604 217.0498	FI	Icariside II
M22	4.93	300	543.1500	–0.6	C_27_H_26_O_12_	565.1453 543.1500 **367.1172**	541.1345 **365.1033** 175.0222 113.0254	BI	Dehydrogenated icaritin-*O*-gluA
M23	4.97	273	387.1445	0.3	C_21_H_22_O_7_	**387.1445 **369.1343 313.0715	385.1286	UFI	Hydrated icaritin
M24	5.02	300	543.1505	0.4	C_27_H_26_O_12_	543.1505 **367.1134 **313.0720	541.1346 **365.1032** 175.0223 113.0255	BI	Dehydrogenated icaritin-*O*-gluA
M25^#^	5.08	270	355.1180	–0.6	C_20_H_18_O_6_	**355.1180** 299.0582	**353.1036** 309.0409 297.0373 281.0481	UFI	Desmethylicaritin
M26	5.13	271	545.1664	0.9	C_27_H_28_O_12_	567.1540 545.1664 **369.1343** 313.0720 299.0585	543.1504** 367.1180** 352.0942 297.0391	BI	Icaritin-*O*-gluA
M27	5.37	268	385.1288	0.3	C_21_H_20_O_7_	407.1136 **385.1288 **367.1186 313.0715	**383.1175** 365.1035 175.0226	UFI	Hydroxylated icaritin
M28	5.54	268	385.1285	–0.5	C_21_H_20_O_7_	**385.1285 **367.1181 313.0719	**383.1170** 365.1046	UFI	Hydroxylated icaritin
M29	5.66	n.a.	385.1290	0.9	C_21_H_20_O_7_	**385.1290 **367.1176 313.0712	**383.1172** 367.1043 175.0262	UFI	Hydroxylated icaritin
M30	6.36	272	367.1180	–0.5	C_21_H_18_O_6_	**367.1180** 352.0948 313.0721 297.0395 253.0496	**365.1047** 351.0765 175.0223 113.0250	FI	Dehydrogenated icaritin

M0, parent drug; M1~M34, metabolites; n.a., not available; P, plasma; U, urine; B, bile; F, feces; I, intestine. gluA, glucuronide conjugates; glc, glucose; rha, rhamnose; xyl, xylose.

# means that the metabolites are exactly identified with reference standards.

**Table 2:** Pharmacokinetic parameters of icaritin in rat plasma after oral administration.

Parameters	100 mg/kg
*T*_max_ (h)	5.3 ± 1.1
*C*_max_ (ng/mL)	294.5 ± 22.7
*t*_1/2_ (h)	8.3 ± 1.0
AUC_0−*t*_ (ng·h/mL)	3048.5 ± 289.0
AUC_0−*∞*_ (ng·h/mL)	3145.0 ± 302.3
MRT_0−*t*_ (h)	9.6 ± 1.1
MRT_0−*∞*_ (h)	10.9 ± 1.3

**Table 3:** Kinetic parameters of icaritin glucuronidation by RLM and RIM (mean ± SD).

Protein source	Metabolite	*V*_max_ (nmol/min/mg)	*K*_m_ (*μ*M)	*K*_i_ (*μ*M)	CL_int_ (mL/min/mg)	Model
RLM	M13	4.06 ± 0.70	3.62 ± 0.99	11.31 ± 3.51	1.12 ± 0.36	SI
	M18	2.39 ± 0.26	1.53 ± 0.34	17.07 ± 4.38	1.56 ± 0.38	SI
RIM	M13	11.88 ± 0.60	8.22 ± 0.92	N.A.	1.45 ± 0.18	MM
	M18	8.23 ± 0.63	9.56 ± 1.51	N.A.	0.86 ± 0.15	MM

*Note.* SI, substrate inhibition model; MM, Michaelis-Menten model; N.A., not available.
