# Supplementary material for: Metabolite Profiling, Pharmacokinetics, and In Vitro Glucuronidation of Icaritin in Rats by Ultra-Performance Liquid Chromatography Coupled with Mass Spectrometry
Source: J Anal Methods Chem. 2017 Jul 10;2017:1073607. doi: 10.1155/2017/1073607 (PMC5529662; doi:10.1155/2017/1073607)
Supplement: Supplementary file 1 — Table S1: Leaner range and LLOQ test of icaritin in rat plasma. Table S2: Matrix effect and recovery test of icaritin in rat plasma (n = 6). Table S3: Intra- and inter-day precision and accuracy test of icaritin in rat plasma. Table S4: Stability test of icaritin in rat plasma under different condition (n = 3). Figure S1: (+) ESI-MS and MS/MS spectra of M0~M30. Figure S2: EICs of M0~M30 in rat intestine samples. Figure S3: Specificity test of icaritin in rat plasma. [file 1073607.f1.doc]

**Supplemental Information**

Metabolite profiling, pharmacokinetics and *in vitro* glucuronidation of icaritin in rats by ultra-performance liquid chromatography coupled with mass spectrometry

Beibei Zhang, Shuzhang Du*, Xiaoli Chen, Rui Zhang, Fangfang Zheng, Xiaojian Zhang*

Department of Pharmacy, the First Affiliated Hospital of Zhengzhou University, Zhengzhou, Henan, 450052, China

* **Correspondence** should be addressed to Pof. Shuzhang Du, [dushuzhang911@163.com](mailto:dushuzhang911@163.com) and Pof. Xiaojian Zhang, [zhangxiaojian_yxb@163.com](mailto:zhangxiaojian_yxb@163.com)

**Table List**

**Table S1** Leaner range and LLOQ test of icaritin in rat plasma.

**Table S2** Matrix effect and recovery test of icaritin in rat plasma (n=6)

**Table S3** Intra- and inter-day precision and accuracy test of icaritin in rat plasma

**Table S4** Stability test of icaritin in rat plasma under different condition (n=3)

**Figure Caption**

**Figure S1** (+) ESI-MS and MS/MS spectra of prototypes and several metabolites.

(a) M0; (b) M15 and M23; (c) M25; (d) M30; (e) M27, M28 and M29; (f) M21; (g) M14 and M16; (h) M17; (i) M19; (j) M20; (k) M13, M18 and M26; (l) M1 and M8; (m) M2 and M10; (n) M3 and M6; (o) M4, M5, M7, M9 and M11; (p) M12, M22 and M24;

**Figure S2** EICs of individual metabolites in rat small intestine samples.

(a) M1, M2, M4, M5, M7-M11, M13, M21;

(b) M3, M6, M12-M14, M16, M18, M22, M24, M26;

(c) M17, M19-M21;

(d) M15, M23, M25, M27-M30;

**Figure S3** Specificity test of icaritin in rat plasma.

(a) blank plasma;

(b) blank plasma spiked with LLOQ solution;

(c) 4 h plasma after oral administration of icaritin;

**Table S1** Leaner range and LLOQ test of icaritin in rat plasma

| Day | Calibration curve | *r*^2^ | Leaner range (ng/mL) | LLOQ (ng/mL) |
| --- | --- | --- | --- | --- |
| 1 | Y=6.55X+2.73 | 0.9926 | 2.0 ~ 512.0 | 2.0 |
| 2 | Y=6.48X+5.10 | 0.9939 |
| 3 | Y=6.74X+1.25 | 0.9973 |

**Table S2** Matrix effect and recovery test of icaritin in rat plasma (n=6)

| Concentration (ng/mL) | ME (%) | RSD (%) | Recovery (%) | RSD (%) |
| --- | --- | --- | --- | --- |
| 4.0 | 94.6±5.5 | 5.9 | 98.9±2.1 | 2.2 |
| 64.0 | 98.7±8.2 | 8.4 | 99.3±1.9 | 2.0 |
| 256.0 | 104.6±8.9 | 8.6 | 99.5±3.2 | 3.3 |

**Note:** ME means matrix effect.

**Table S3** Intra- and inter-day precision and accuracy test of icaritin in rat plasma

| Conc.  (ng/mL) | Intra-day (n=6) | | | Inter-day(n=18) | | |
| --- | --- | --- | --- | --- | --- | --- |
|  | Measured  Conc. (ng/mL) | RE (%) | RSD (%) | Measured  Conc. (ng/mL) | RE (%) | RSD (%) |
| 2.0 | 2.1±0.4 | 14.7 | 17.4 | 2.1±0.3 | 15.3 | 15.6 |
| 4.0 | 4.1±0.5 | 8.2 | 13.2 | 4.1±0.3 | 6.8 | 7.4 |
| 64.0 | 68.5±5.3 | -11.7 | 7.8 | 66.7±4.4 | -12.2 | 6.6 |
| 256.0 | 262.8±29.7 | 7.6 | 11.3 | 266.8±27.3 | 5.3 | 10.2 |

**Note:** Conc. means concentration.

**Table S4** Stability test of icaritin in rat plasma under different condition (n=3)

| Conc.  (ng/mL) | At 25 °C  for 8 h | | Post-preparation for 12 h | | 3 Freeze-thaw  cycles | | At -80 °Cfor 60 h | |
| --- | --- | --- | --- | --- | --- | --- | --- | --- |
|  | RE (%) | RSD (%) | RE (%) | RSD (%) | RE (%) | RSD (%) | RE (%) | RSD (%) |
| 4.0 | -12.3 | 5.6 | 2.9 | 2.9 | -12.9 | 4.9 | -12.6 | 11.3 |
| 64.0 | 12.4 | 3.2 | 12.5 | 6.5 | 13.8 | 1.2 | 11.6 | 4.3 |
| 256.0 | 7.4 | 2.5 | 11.7 | 4.1 | 10.8 | 2.3 | 12.8 | 7.1 |

**Note:** Conc. means concentration.


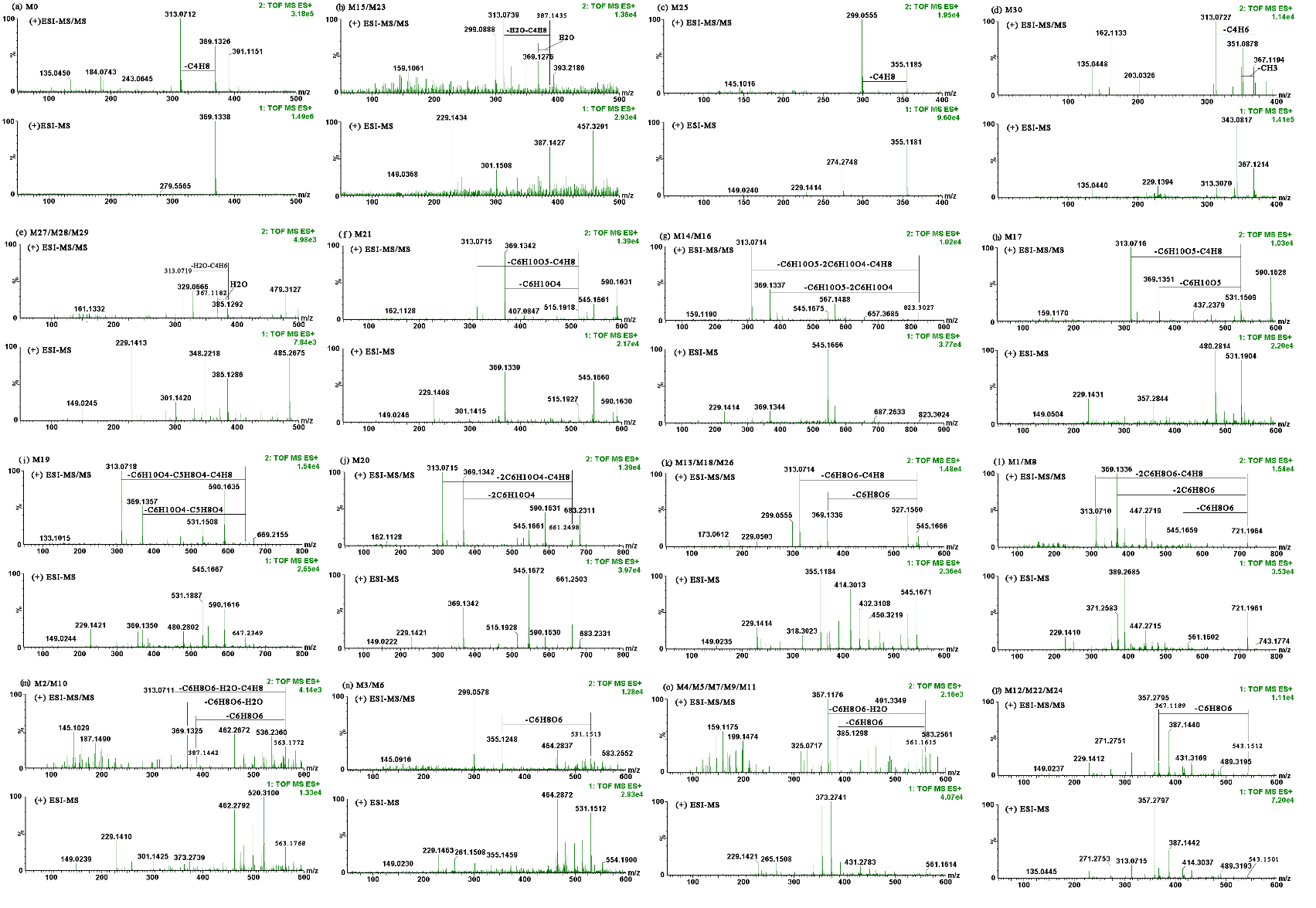


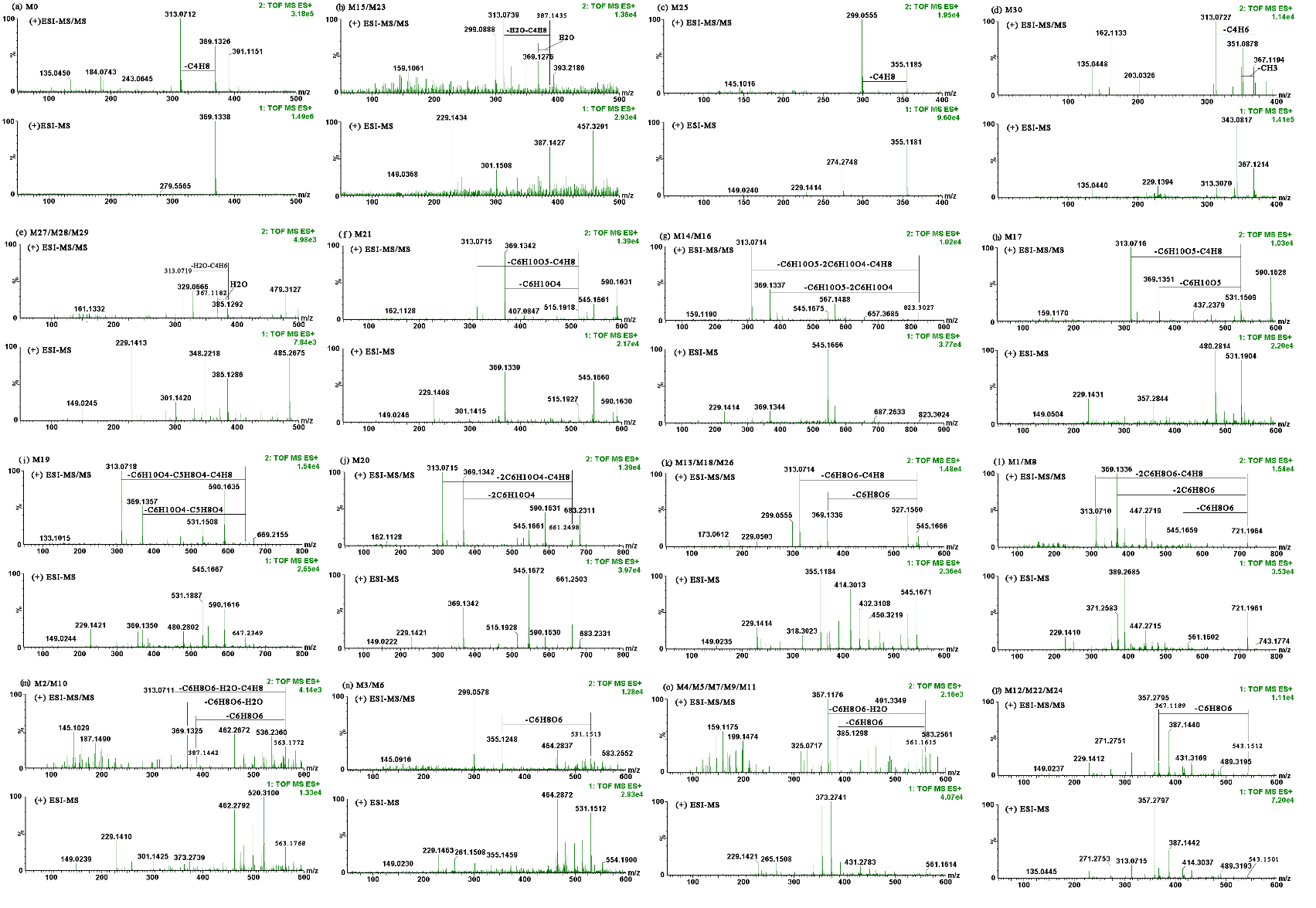


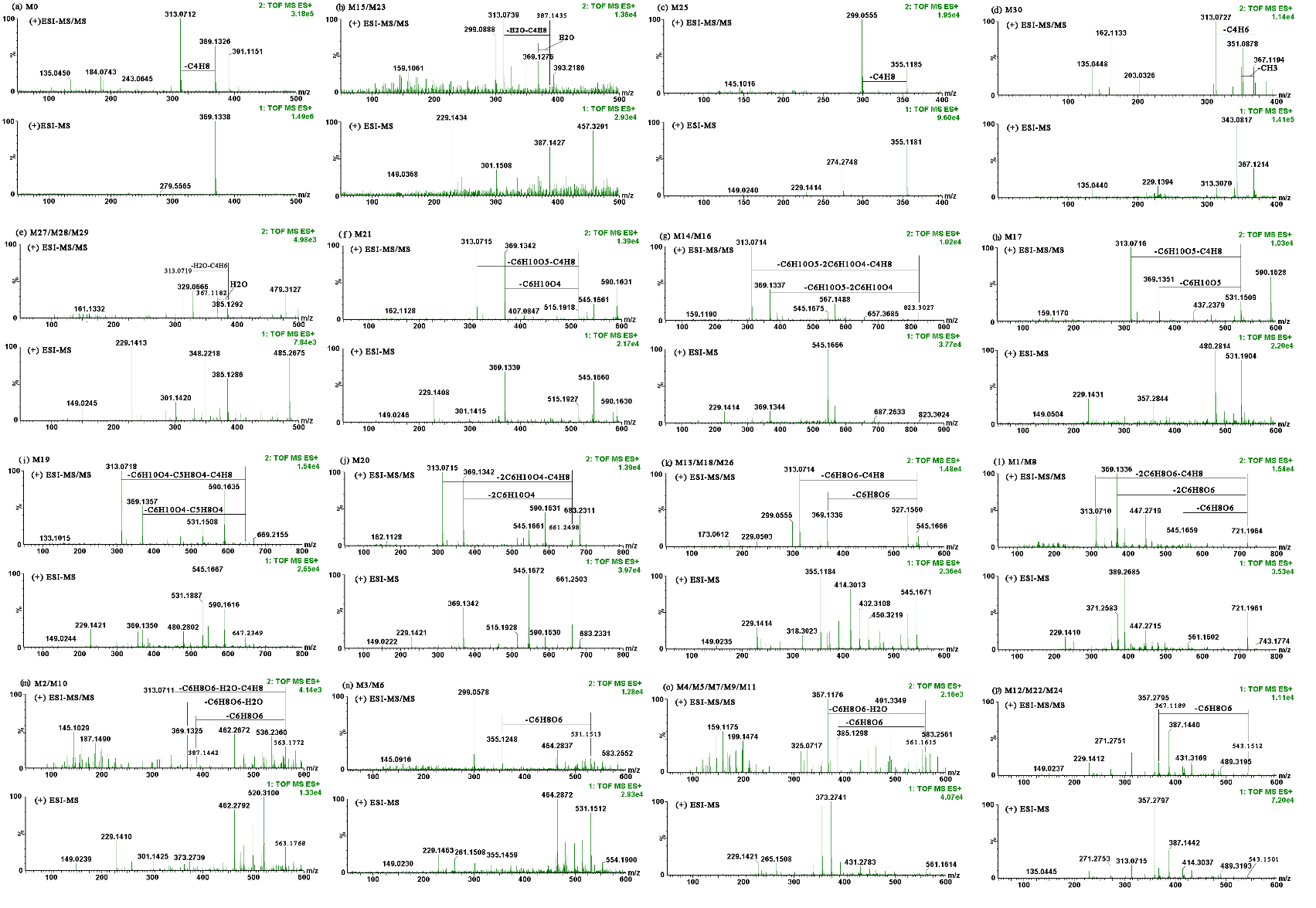


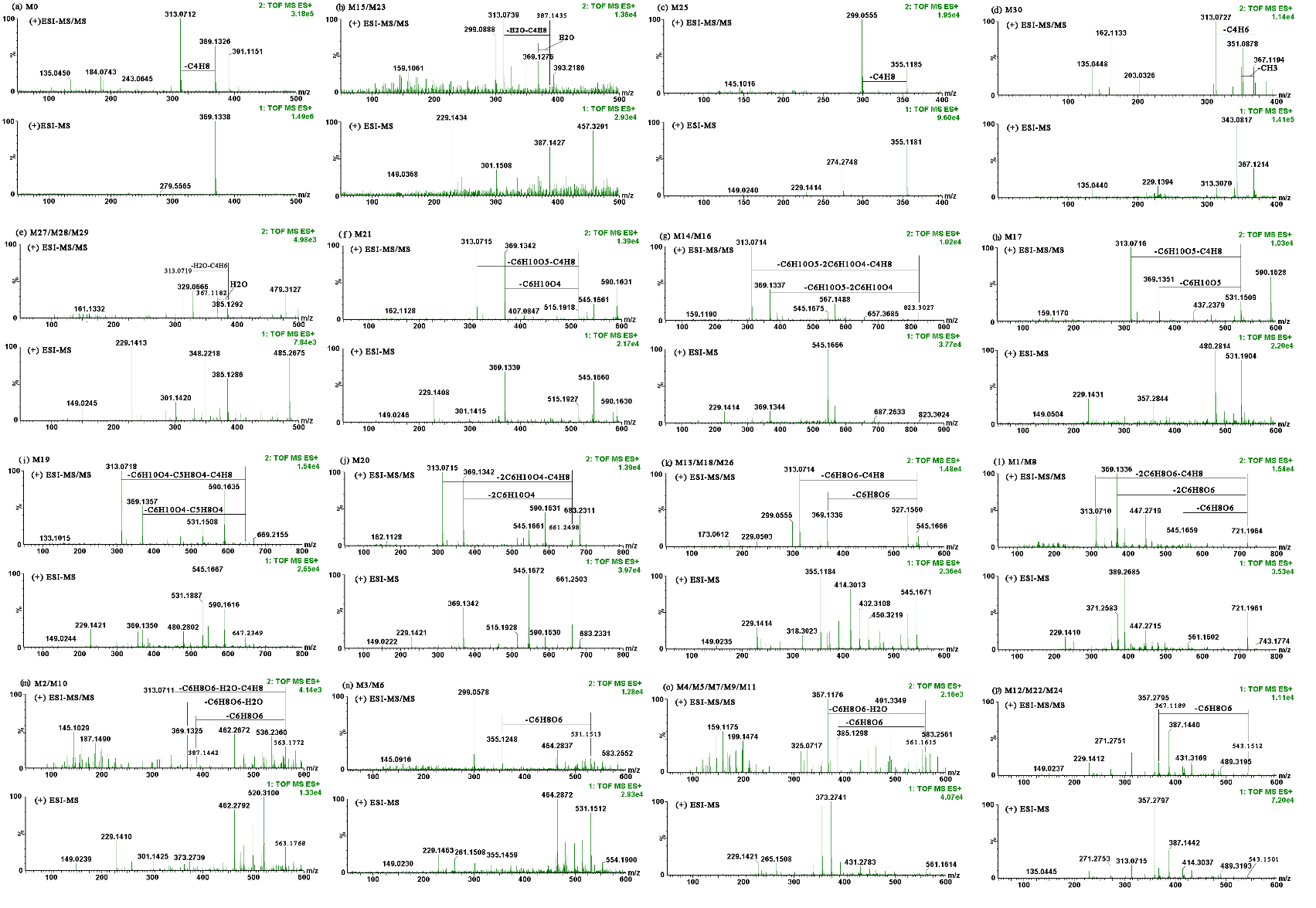

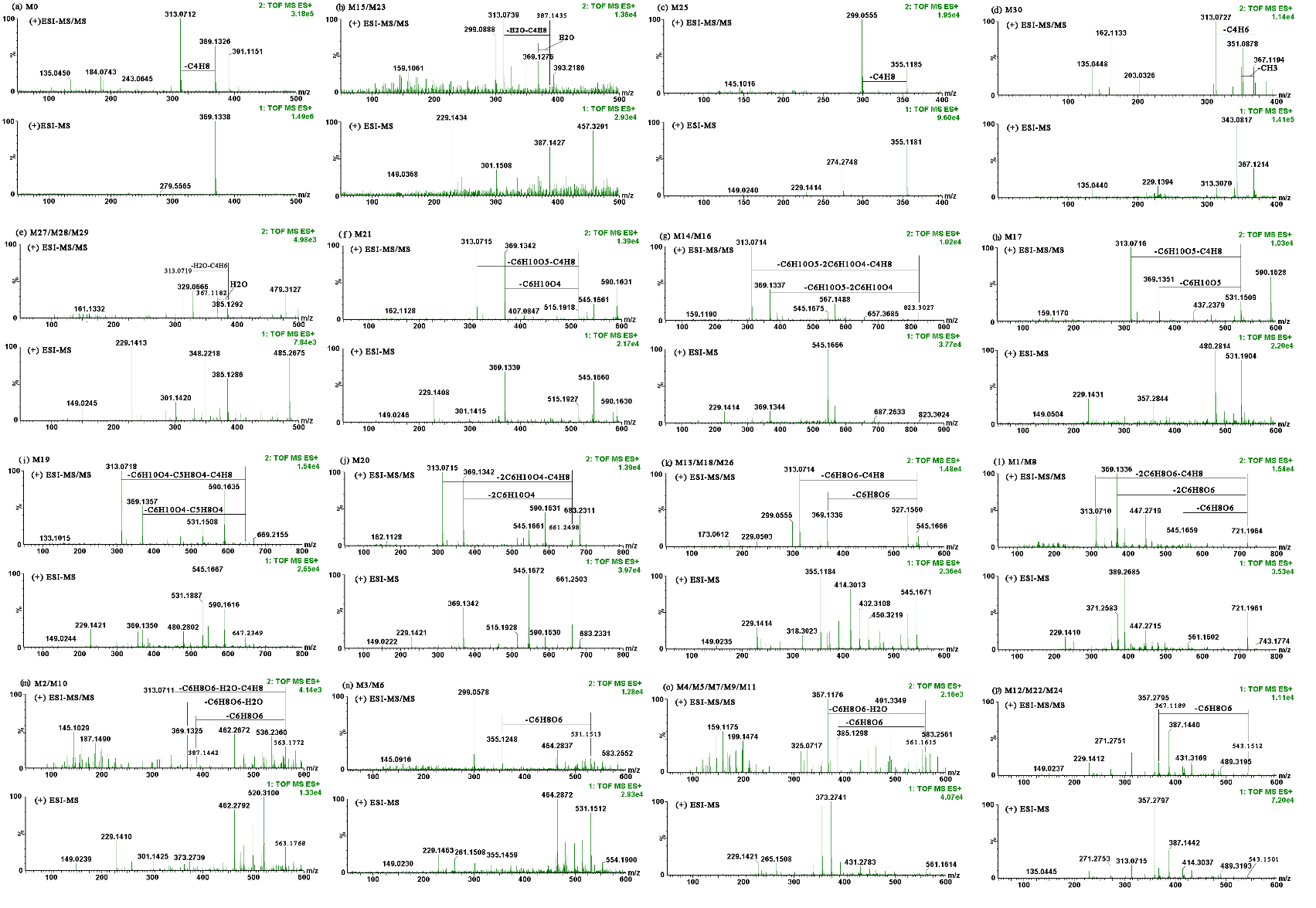


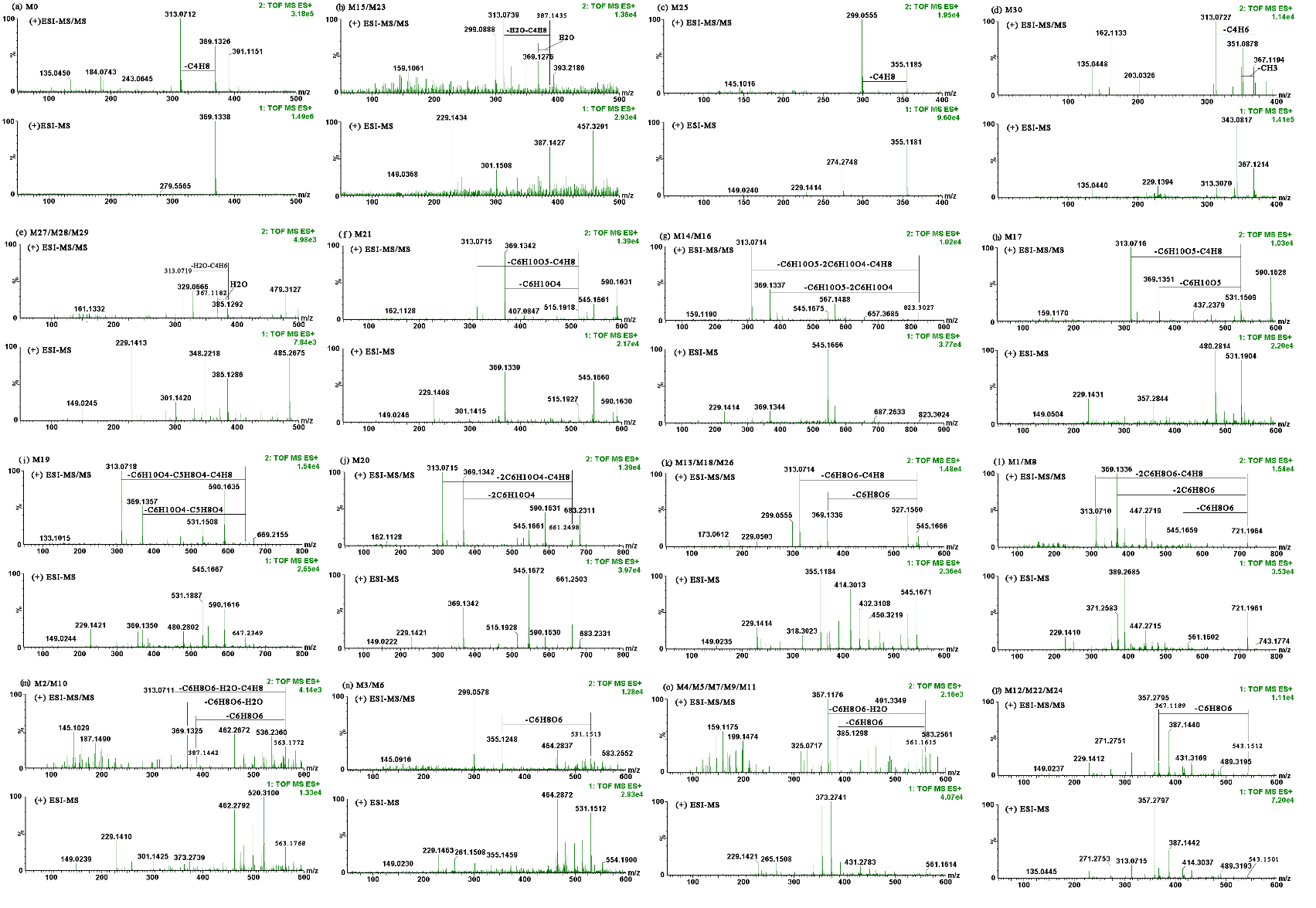


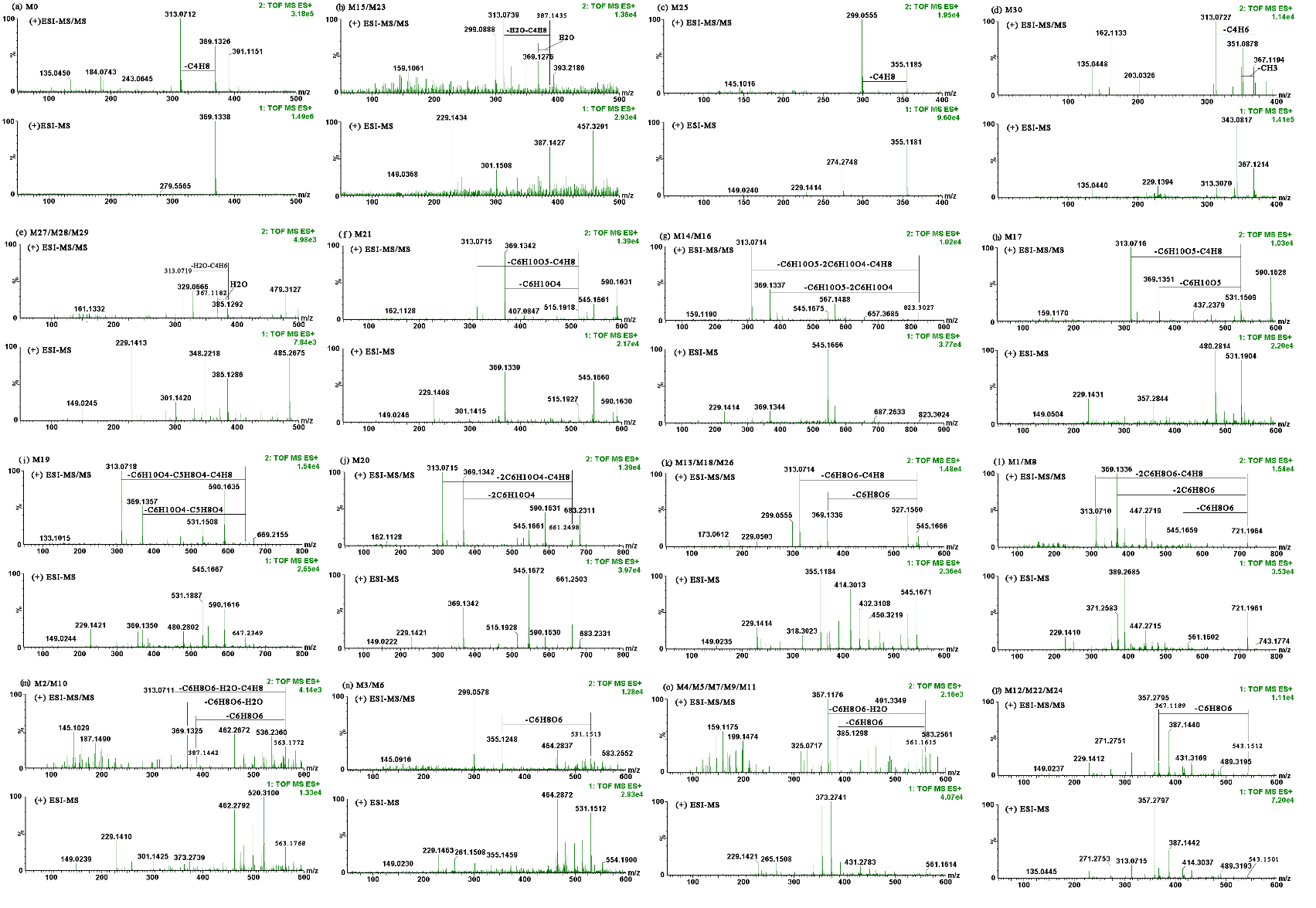


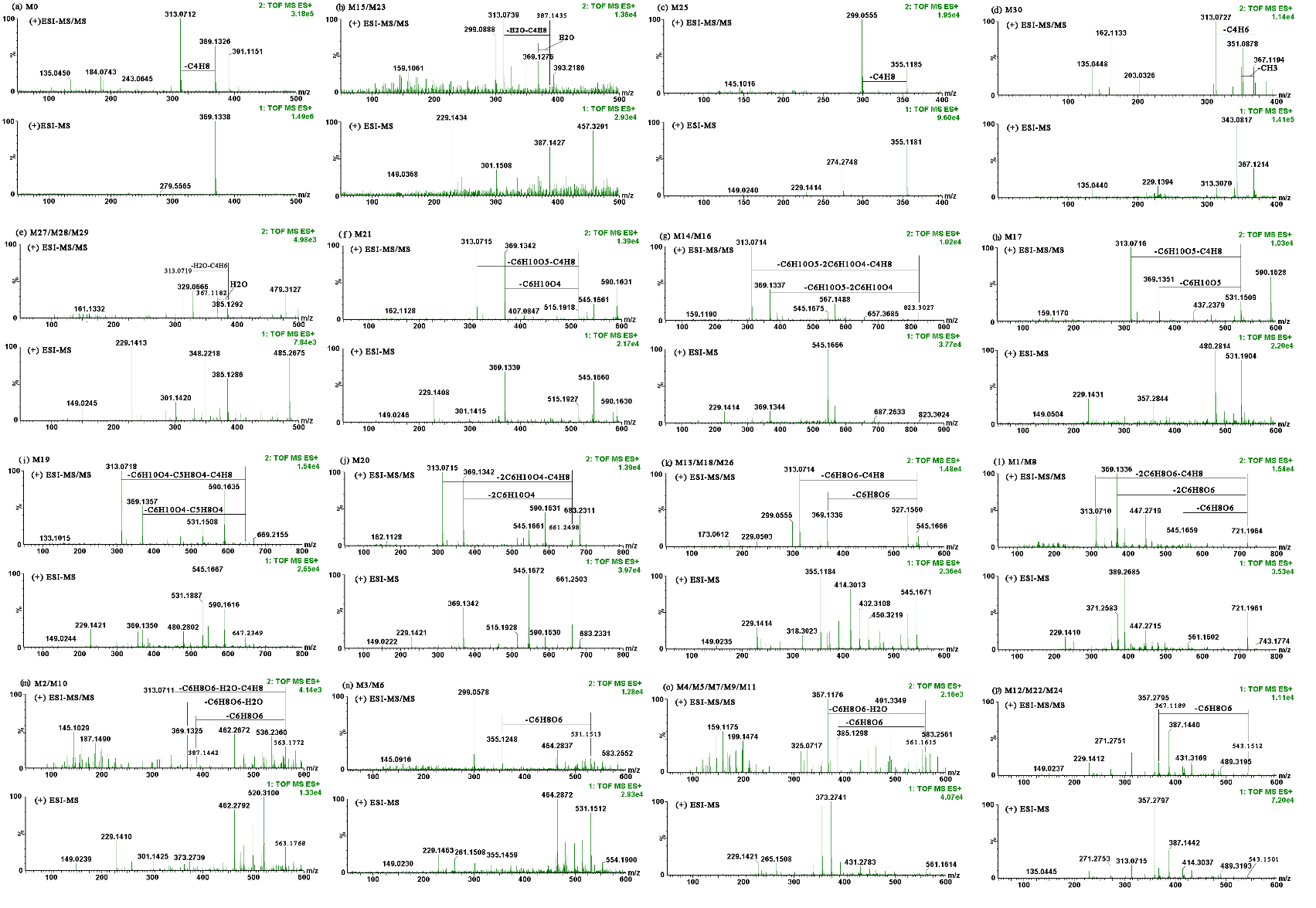


**Figure S1** (+) ESI-MS and MS/MS spectra of prototypes and several metabolites.

(a) M0; (b) M15 and M23; (c) M25; (d) M30; (e) M27, M28 and M29; (f) M21; (g) M14 and M16; (h) M17; (i) M19; (j) M20; (k) M13, M18 and M26; (l) M1 and M8; (m) M2 and M10; (n) M3 and M6; (o) M4, M5, M7, M9 and M11; (p) M12, M22 and M24;


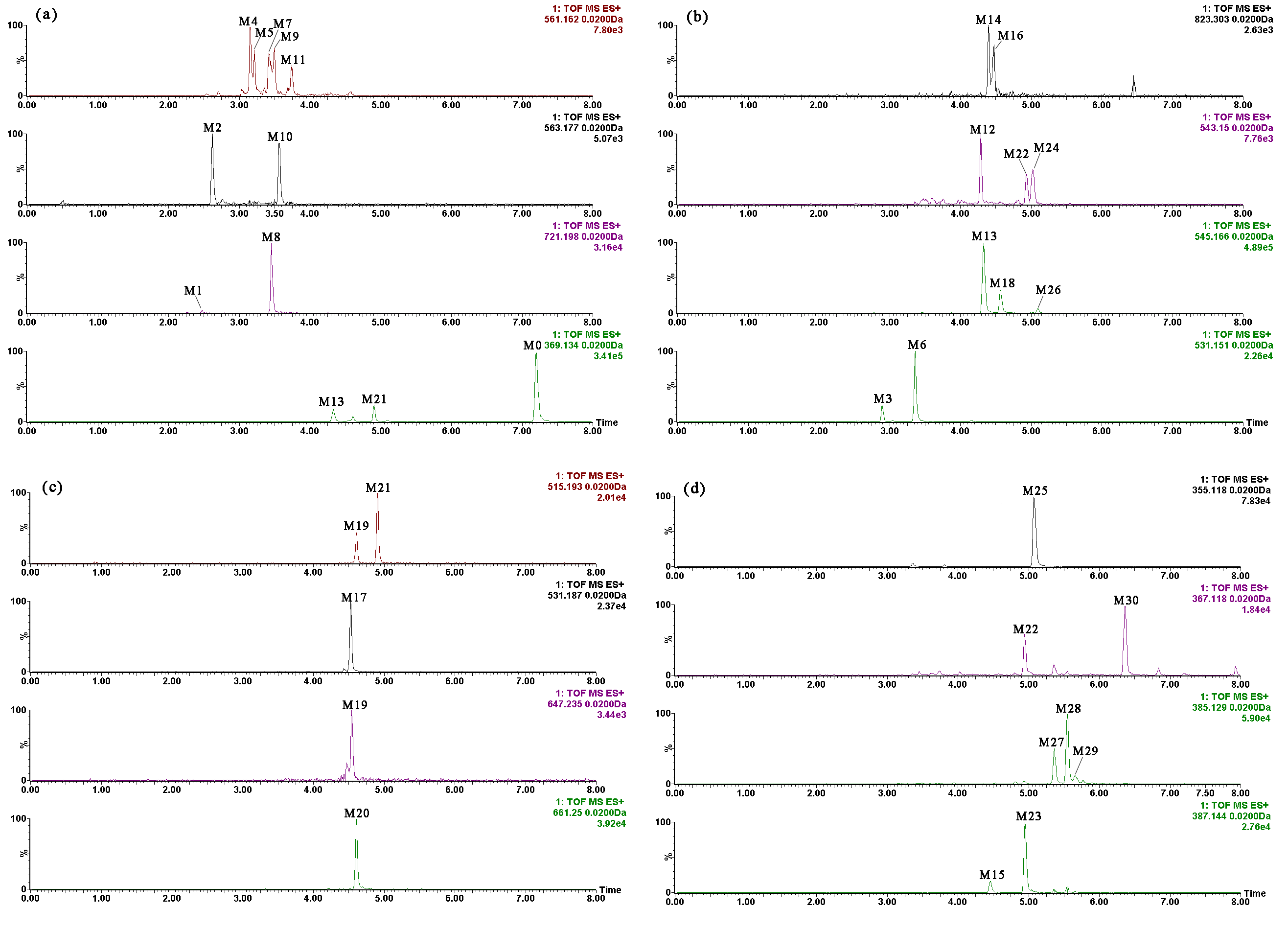


**Figure S2** EICs of individual metabolites in rat small intestine samples.

(a) M1, M2, M4, M5, M7-M11, M13, M21;

(b) M3, M6, M12-M14, M16, M18, M22, M24, M26;

(c) M17, M19-M21;

(d) M15, M23, M25, M27-M30;


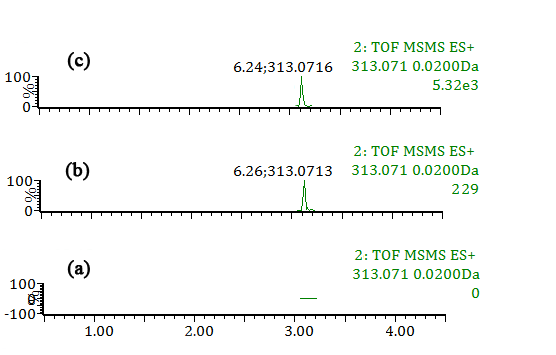


**Figure S3** Specificity test of icaritin in rat plasma.

(a) blank plasma;

(b) blank plasma spiked with LLOQ solution;

(c) 4 h plasma after oral administration of icaritin;
